# Evaluation of a New Method of Fossil Retrodeformation by Algorithmic Symmetrization: Crania of Papionins (Primates, Cercopithecidae) as a Test Case

**DOI:** 10.1371/journal.pone.0100833

**Published:** 2014-07-03

**Authors:** Melissa Tallman, Nina Amenta, Eric Delson, Stephen R. Frost, Deboshmita Ghosh, Zachary S. Klukkert, Andrea Morrow, Gary J. Sawyer

**Affiliations:** 1 Department of Biomedical Sciences, Grand Valley State University, Allendale, Michigan, United States of America; 2 New York Consortium of Evolutionary Primatology Morphometrics Group, New York Consortium of Evolutionary Primatology, New York, New York, United States of America; 3 Department of Computer Science, University of California Davis, Davis, California, United States of America; 4 Department of Anthropology, Lehman College of the City University of New York, Bronx, New York, United States of America; 5 New York Consortium in Evolutionary Primatology, New York, New York, United States of America; 6 PhD Program in Anthropology, City University of New York Graduate Center, New York, New York, United States of America; 7 Department of Vertebrate Paleontology, American Museum of Natural History, New York, New York, United States of America; 8 Department of Anthropology, University of Oregon, Eugene, Oregon, United States of America; 9 Stratovan Corporation, Sacramento, California, United States of America; 10 Department of Biology, Grand Valley State University, Allendale, Michigan, United States of America; 11 Department of Anthropology, American Museum of Natural History, New York, New York, United States of America; Max Planck Institute for Evolutionary Anthropology, Germany

## Abstract

Diagenetic distortion can be a major obstacle to collecting quantitative shape data on paleontological specimens, especially for three-dimensional geometric morphometric analysis. Here we utilize the recently -published algorithmic symmetrization method of fossil reconstruction and compare it to the more traditional reflection & averaging approach. In order to have an objective test of this method, five casts of a female cranium of *Papio hamadryas kindae* were manually deformed while the plaster hardened. These were subsequently “retrodeformed” using both algorithmic symmetrization and reflection & averaging and then compared to the original, undeformed specimen. We found that in all cases, algorithmic retrodeformation improved the shape of the deformed cranium and in four out of five cases, the algorithmically symmetrized crania were more similar in shape to the original crania than the reflected & averaged reconstructions. In three out of five cases, the difference between the algorithmically symmetrized crania and the original cranium could be contained within the magnitude of variation among individuals in a single subspecies of *Papio*. Instances of asymmetric distortion, such as breakage on one side, or bending in the axis of symmetry, were well handled, whereas symmetrical distortion remained uncorrected. This technique was further tested on a naturally deformed and fossilized cranium of *Paradolichopithecus arvernensis.* Results, based on a principal components analysis and Procrustes distances, showed that the algorithmically symmetrized *Paradolichopithecus* cranium was more similar to other, less-deformed crania from the same species than was the original. These results illustrate the efficacy of this method of retrodeformation by algorithmic symmetrization for the correction of asymmetrical distortion in fossils. Symmetrical distortion remains a problem for all currently developed methods of retrodeformation.

## Introduction

Among the main contributions to the study of evolution by paleontology is the analysis of fossils, which provide dated records of the morphological pathways evolution has actually taken. One –of the challenges with the study of fossils is that they generally have been subjected not only to trauma during life but also to various forms of diagenesis, including breakage, shear, and warping, after death. Geological compaction during the process of fossilization causes “flattening” and “bending” of the bones, which in the case of midline elements results in loss of their bilateral symmetry. This change in shape presents a challenge to researchers seeking to collect quantitative data – and, in particular, three dimensional shape data - from fossils and to compare them with other specimens in analyses of functional morphology, phylogeny, ontogeny, and other questions. Thus, it is desirable to reconstruct the antemortem shape of any deformed fossils before conducting further studies.

The operation of reconstructing antemortem shape from a deformed specimen is called “retrodeformation” (a term apparently first used by Williams [1]), while we will call the more specific operation of restoring symmetry “symmetrization” [2],[3]. Symmetrization is used as a step in nearly all current methods of retrodeformation [4], [5], [6],[7], [8], [9], and the choice of symmetrization technique may affect the shape and size of the result**;** certainly there are an infinite number of (retro)deformations that can symmetrize a given fossil. These and other methods of retrodeformation have been applied in recent years to answer questions about a wide variety of fossil taxa, for example, sauropod dinosaurs *e.g.* [10], therapsids *e.g.* [11], and hominins *e.g.* [12], [13], [14].

The current standard technique for restoring bilateral symmetry is to reflect the landmarks across the sagittal plane, calculate the average of each landmark with its reflection, and then warp the original untransformed shape to the averaged landmarks. The best variant of this approach begins by reflecting the specimen through an arbitrary plane and then aligning all mid-sagittal and bilaterally symmetrical pairs of landmarks between the original and reflected specimens [7]. Gunz et al. [8] used this form of “reflection & averaging” to reverse moderate amounts of synthetically introduced uniform shear, which it does well. But reflection & averaging does not reverse the effects of either bending or compression; for example, reflection & averaging will not restore any height to a specimen that has been supero-inferiorly compressed or any breadth to a specimen that has been compressed medio-laterally. In addition, Angielcyk and Sheets [15] found that reflection & averaging did not accurately restore specimens that were deformed using computer simulations.

Motani [6] and Zollikofer and Ponce de Leon [4] have considered symmetrization assuming that the taphonomic deformation is an affine compression, that is, a uniform transformation of the specimen in which distances in one specific direction are made uniformly smaller, while distances in directions orthogonal to this axis remain unchanged. Reversing compression, by stretching, has the potential to restore a specimen to its original size (depending on the direction of the compression; a fossil that experiences a perfectly supero-inferiorly oriented compression will remain symmetrical and cannot be restored to its original shape by symmetrization). Unfortunately, even if given a perfectly symmetrical landmark set which has experienced a perfectly uniform compression, there are still an infinite number of possible directions in which the landmark set can be stretched in order to produce a perfectly symmetrical result [16]. Additionally, for any fixed direction of stretch, there is a unique amount of stretch that symmetrizes the landmark set. Given that in any real situation, the original individual was not perfectly symmetrical, the goal is to find the “best” uniform stretch which produces an output that minimizes the deviation from symmetry. Subsol et al. [17] and Motani [6] both chose to stretch in the direction leading to the minimal deviation from symmetry in the output landmark set. Zollikofer and Ponce de Leon [4] instead chose the direction that symmetrizes with the smallest stretch. Ogihara et al. [5] proposed a non-linear method for retrodeformation. They sought to minimize the difference between the deformed and undeformed landmark positions while symmetrizing the specimen, combining three steps, each with its own exact least-squares solution. This approach does not assume that stretching is necessary. Our non-linear symmetrization method [9], summarized below, combines the stretching approach of Zollikofer and Ponce de Leon [4] with an interpolation technique that handles specimens that have undergone bending as well as compression.

Moreover, none of the prior attempts at restoring symmetry (with the exception of Gunz et al [8]) included any means of evaluating the accuracy of the retrodeformation [4], [5] [6]. Nonetheless, testing methods of cranial reconstruction, especially for fossils, does have a long history in paleoanthropology. Almost exactly 100 years ago, Arthur Keith wished to demonstrate that he could successfully reconstruct the Piltdown cranium from its fragments. According to Spencer [18], some of Keith’s colleagues broke a modern cranium into fragments roughly corresponding to those recovered at Piltdown and gave them to Keith, who reconstructed them and measured the cranial capacity within a few cm^3^ of the undamaged specimen. This result was reported to the Royal Anthropological Institute on January 20, 1914 and published as Keith [19]. We seek to follow in this tradition by mechanically (as opposed to virtually) deforming known and measured cranium, and gauging our method against that standard.

The purpose of this paper is to test our method of fossil symmetrization (as described in Ghosh et al. [9] and below). Our methods are twofold: first using artificially deformed casts of a cranium of a female individual of *Papio hamadryas kindae* of known shape. Second, as our experimental deformations cannot reproduce exactly what happens during the complex geological and taphonomic processes of diagenesis experienced by real specimens, we also apply this technique to a fossil cranium of a male individual of the Pleistocene cercopithecine primate *Paradolichopithecus arvernensis*, which -exhibits asymmetrical deformation.

## Materials and Methods

### Process of artificial deformation

**Figure 1:**
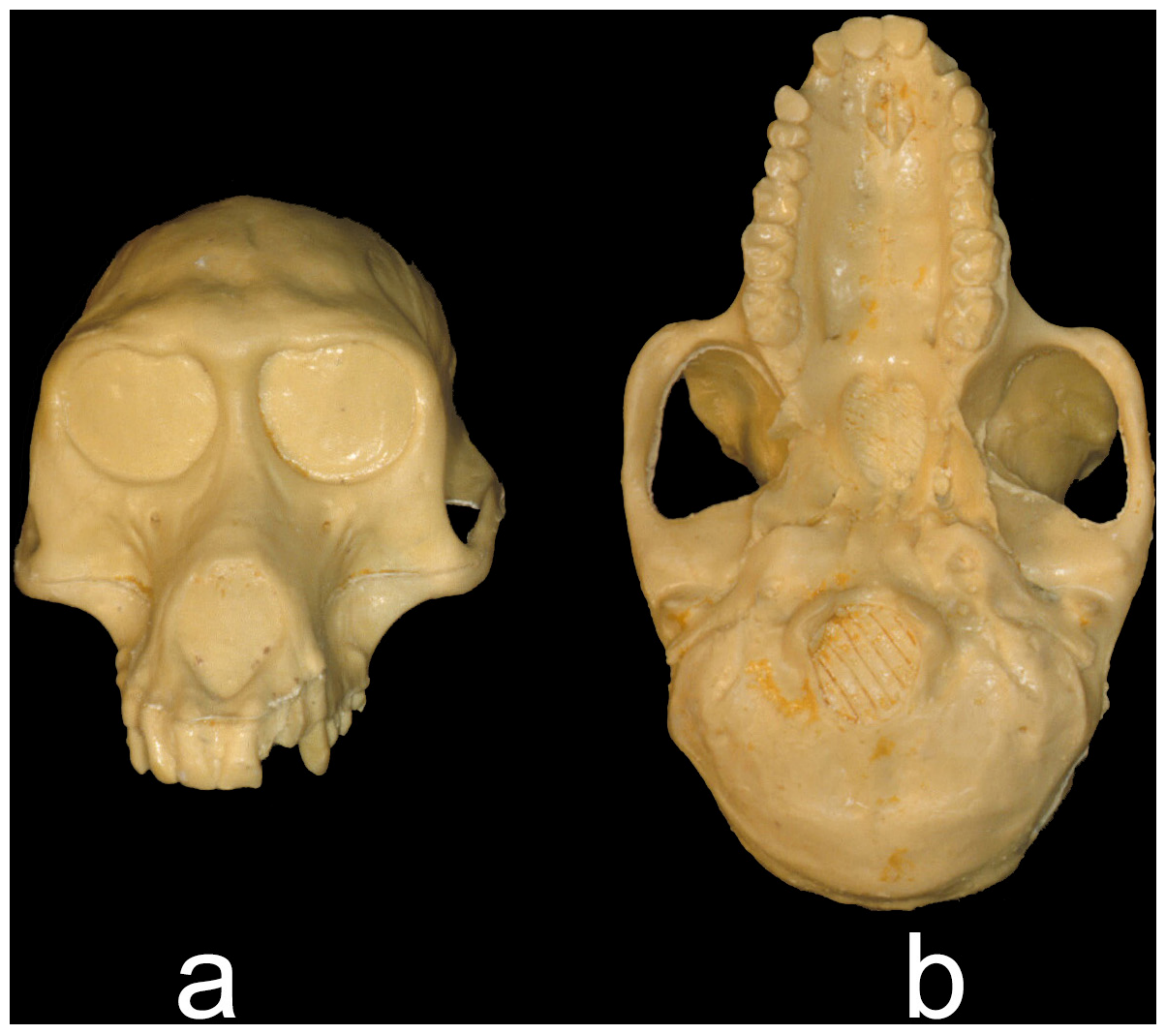
Undeformed cast of NHML ZD.1961.776 in (a) anterior and (b) inferior views.

In order to rigorously evaluate our method of retrodeformation, and following the spirit of Keith’s experiment, we wanted to apply our method to actual papionin morphology using a known specimen where we could measure how accurately the original morphology was restored. A flexible mold was made (by G.J.S.) of the facial and basal region of a cast of a female cranium of *Papio hamadryas kindae* (Natural History Museum London, Zoology Department, [NHML ZD] 1961.776, Figure 1) in “Dragonskin” silicone rubber (Smooth-On Corp.). This material is resistant to the tearing common to silicone rubber molding compounds, allowing the mold to be twisted and squeezed without damage; the mold was made in one piece in order to avoid the need to fit two sides together after deformation. A hard plaster (Hydrocal white gypsum cement, CAS 26499-65-0) was prepared with water and poured into the mold, which was immediately deformed by squeezing or twisting it with one or both hands while the plaster was still wet; this position was held for approximately 5–10 minutes until the plaster set. The manual deformation was designed to mimic the varying levels of deformation present in the fossil record. Each deformed cast was allowed to harden for 5–12 hours before it was removed from the mold. In all, five deformed versions of the *P. h. kindae* cranium were produced (by G.J.S. and Z.S.K.) and designated “Cranium 1” through “Cranium 5”. They represent deformations that range in both degree (from light to heavy) and pattern (including symmetrical and asymmetrical). For example, in some natural cases the diagenetic deformation is mostly asymmetrical, in the form of a shear (*e.g*., the cranium of *Sahelanthropus* [20]), or crushing and breakage on one side (*e.g.,* the left side of the KNM-RU 2036 *Proconsul* cranium [21]); in other cases the diagenetic deformation is more symmetrical, and the specimen is compressed in a single direction (*e.g.,* the skeleton of *Oreopithecus* as an extreme example [22]). Our goal was to examine the efficacy of our algorithmic symmetrization method in ways that could apply to real-life situations. The deformed casts were subsequently scanned using a Breuckmann Opto-top HE imaging system to generate 3D surface models.

### Retrodeformation by algorithmic symmetrization

Algorithmic symmetrization was performed in Landmark Editor [23] using the retrodeformation plug-in [9], which restores the bilateral symmetry of an input shape by stretching each local region to correct for affine deformation and then combining those locally symmetric regions into a bilaterally symmetric shape. Each local region is defined by a set of corresponding landmarks chosen by the user across the local midsagittal plane. A user can define as many pairs of symmetrical landmarks as can be reliably identified; to retrodeform the test crania in this study via algorithmic symmetrization, 40–45 bilateral landmark points were used on each cranium, which was the maximum number of bilateral landmarks that could be precisely collected. The retrodeformation by algorithmic symmetrization protocol differed from cranium to cranium, and depended on which bilaterally symmetrical points could be assessed most accurately in each individual case.

As described in detail in Ghosh et al. [9], in the first step of the symmetrization algorithm, we correct for “flattening” of the shape by finding, for each bilateral landmark pair, a minimal stretch that makes the neighborhood around that pair symmetrical across its local midsagittal plane. The size of the neighborhood is a parameter that can be modified, but we use the default value in the software for all of these experiments. In the second step, we minimally rotate each local plane of symmetry to coincide with the global midsagittal plane. Finally, we solve for landmark positions that are symmetrical around this global midsaggital plane and for which the inter-landmark vectors match those in the locally symmetrized neighborhoods as well as possible, in a least-squares sense. After algorithmic symmetrization was completed, the shape was further symmetrized by averaging it with its reflected model, following the method of Gunz et al [8]. This third step involves reflecting a shape across a plane and using Landmark Editor to generate correspondences between them. The corresponding landmark positions were averaged to define a set of new symmetrical landmarks. The shape is then deformed using a thin-plate spline warp defined by the transformation of these landmark positions. Algorithmic symmetrization of cranium 5 required an extra step in which the left half of the cranium (with greater distortion) was warped 30% of the way to a reflected landmark configuration representing the left side via thin-plate spline deformation using Landmark Editor. This cranium was then symmetrized and reflected in the same manner as crania 1–4. In order to determine whether our algorithmic method of symmetrization performs better than reflection & averaging alone, we also computed models of deformed crania 1–5 using only reflection & averaging in Landmark Editor. The same landmark protocol used for algorithmic symmetrization for each cranium was also used for reflection & averaging. Each cranium was reflected, corresponding landmarks were placed on both the original and reflected crania, and the average shape was computed. The original model was warped to the averaged configuration via thin-plate spline deformation.

### Evaluation of algorithmic symmetrization

**Figure 2:**
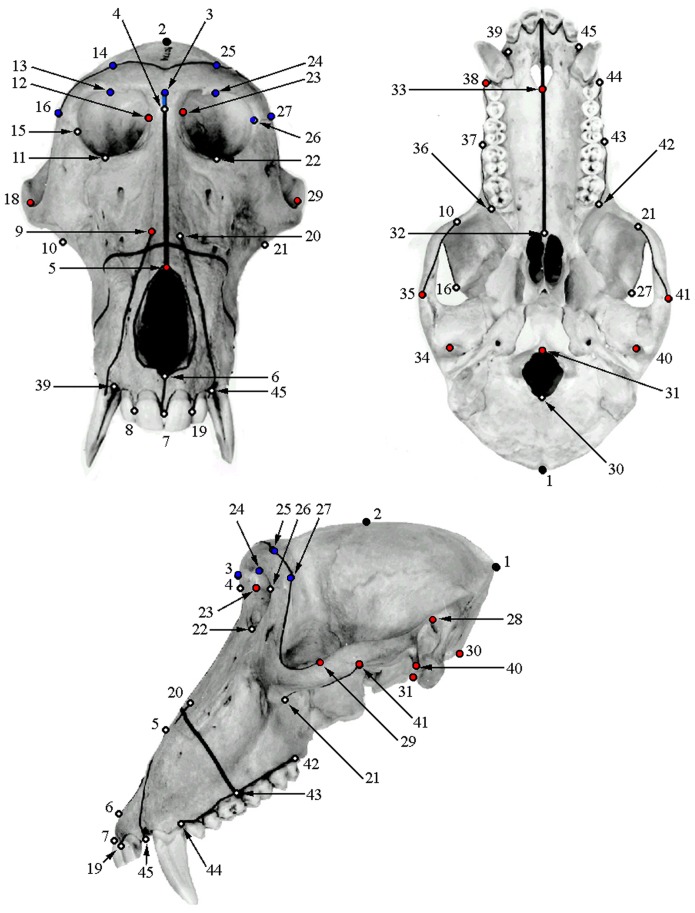
Landmarks used in this study. Blue landmarks indicate those eliminated for analyses using the artificially deformed crania, red landmarks indicate those eliminated for analyses of *Paradolichopithecus,* and black landmarks indicate those eliminated in all analyses.

In order to evaluate the results of the algorithmic symmetrization process, the landmarks and semilandmark curves defined by Frost et al. [24] (Table 1–2, Figure 2) were placed with Landmark Editor on surface scans of the original cranium, the deformed crania, the reflected & averaged crania, and the algorithmically symmetrized crania (landmarks with curves by A.M. for a first analysis, and landmarks only by S.R.F. for a second analysis - see below). This series of landmarks has been demonstrated to capture subtle differences in cranial morphology in papionins [24], [25], [26] [27]. Landmarks and semilandmarks are defined as a series of *x,y,z* coordinates that, when used together, describe a shape in three-dimensional space [28], [29]. Landmark data were used to evaluate our retrodeformations using Procrustes distances to measure differences between shapes and principal component analyses as a dimension reduction technique to visually represent the locations of specimens in morphospace. These results by algorithmic symmetrization were also compared to results from symmetrization by reflection & averaging alone (following [7],[8]). These sets of landmarks were used after both forms of retrodeformation and were not used specifically to create the retrodeformed models.

**Table 1:** List of landmarks used in these analyses.

Number (Right/Left)	Point	Description/Notes
*3.	Glabella	Most anterior point of frontal, as viewed in Frankfurt horizontal.
4.	Nasion	Fronto-nasal suture in midline.
**5.	Rhinion	Most anterior point in midline on nasals (i.e. “end” of the nasals).
6.	Nasospinale	Inferiormost midline point of piriform aperture.
7.	Prosthion	Anteroinferior point on projection of premaxilla between central incisors.
8./19.	Prosthion2	Antero-inferiormost point on pre maxilla, equivalent to prosthion, but between central and lateral incisors.
**9./20.	Premax-Max Superior	Where premaxillo-maxillary suture meets nasal bone, or aperture, if it does not continue to the nasal bone.
10./21.	Zygo-Max Inferior	Anteroinferior point of zygomaticomaxillary suture, in antero-lateral view.
11./22.	Zygo-Max Superior	Anterosuperior point of zygomaticomaxillary suture (taken at orbit rim).
**12./**23.	Dacryon	Junction of frontal, lacrimal and maxilla.
*13./*24.	Mid-Torus Inferior	Point on inferior margin of supraorbital torus (superior margin of orbit) roughly at middle of orbit.
*14./*25.	Mid-Torus Superior	Superior to MTI on superior most point of spraorbital torus when viewed in Frankfurt horizontal (see Line I).
15./*26.	Frontomalare Orbitale	Where frontozygomatic suture crosses the inner orbital rim.
*16./*27.	Frontomalare Temporale	Where frontozygomatic suture crosses lateral edge of zygoma (LEZ) if suture isn’t straight, project course of middle third laterally to LEZ.
**17./**28.	Porion	Top of auditory meatus, helps define Frankfurt Horizontal
**18./**29.	Zygo-Temp Superior	Superior point of zygomatico-temporal suture on lateral face of zygomatic arch.
30.	Opisthion	Posterior most point of foramen magnum.
**31.	Basion	Anterior most point of foramen magnum.
32.	Staphylion	Midline point on palate on linetangent to anteriormost points on choanae.
**33.	Incisivion	Midline point at the anteriormost point of the maxilla ( = posterior end of the incisive foramen),extrapolated if broken or asymmetrical.
**34./**40.	Postglenoid	Tip (or midpoint of area).
**35./**41.	Zygo-Temp Inferior	Inferolateral point of zygomaticotemporal suture on lateral face of zygomatic arch.
36./42.	Distal M3	Distal midpoint projected (laterally) onto alveolar margin.
37./43	M1-2 Contact	Projected (laterally) onto alveolar margin.
**38./44.	Mesial P3	Most mesial point on P3 alveolus, projected onto alveolar margin.
39./45.	Premax-Max Inferior	Where premaxillomaxillary suture crosses alveolar margin.

The numbers correspond to those of Frost et al. (2003) and Figure 2. Landmarks indicated by one asterisk (*) were excluded in analyses of deformed crania, whereas those with two (**) were excluded in analyses of *Paradolichopithecus* crania.

**Table 2:** List of semilandmark curves used in these analyses.

Curve	# Semi-landmarks	Description/Notes
Nasal Aperture	17	From rhinion counterclockwise around nasal aperture, through nasospinale, down right and up left.
Nasospinale – Prosthion	4	Follows midline.
R./L. Premax-Max Suture	11	From Premax-Max Superior to Premax-Max Inferior along suture.
Dorsal Rostrum	23	From right M1-2 contact superiorly across rostrum midline to left M1-2 contact, orthogonal to alveolar plane.
R./L. Orbit	13	Orbital margin from Zygo-Max Superior laterally through Frontomalare Orbitale, medially through Mid-Torus Inferior and hamulus/notch to dacryon.
R./L. Temporal Margin	10	From Frontomalare Temporale to Zygo-Temp Superior along temporal margin.
R./L. Inferior ZygomaticMargin	9	From Zygo-Max Inferior to Zygo-Temp Inferior along inferior-most margin of zygomatic.
R./L. Alveolar Margin	10	Along outer margin of alveolar process from Distal M3 to Mesial P3.

The numbers correspond to those of Frost et al. (2003) and Figure 2.

### Procrustes Distance

Procrustes distances between the original cranium, each deformation, and its subsequent algorithmically symmetrized cranium were calculated based on the full configuration, including landmarks and semilandmark curves (placed by A.M.). Using both landmarks and curves better represents the geometry of the cranium and is a more complete evaluation of shape similarity. Procrustes distance is defined as the sum of squares difference between two optimally superimposed landmark configurations [28]. The Procrustes distance between the original and the deformed specimens gives a metric for the degree of deformation, and the distance between the original and retrodeformed specimens gives a metric for the success of the correction – the better the retrodeformation, the smaller the Procrustes distance between the retrodeformed skull and the original undeformed specimen. Procrustes distances based on only type I, II and III landmarks (placed by S.R.F.) were also calculated, for the purposes of comparing these distances to large published data sets. These sets of Procrustes distances were compared in several ways. First, they were compared relative to the distribution of pairwise Procrustes distances representing intraobserver error. In order to determine the range of intraobserver error, we used data from a previous teaching approach in which 9 different users landmarked a single *Papio hamadryas ursinus* cranium 3 to 13 times each with the same landmark configuration using a Microscribe (Table 3). Procrustes distances within the range of expected intraobserver error would indicate that the retrodeformed cranium is close to or indistinguishable from the original. Second, the Procrustes distances between the original and retrodeformed crania were compared to both a large sample of adult *Papio,* encompassing five extant subspecies [24] and a sample of adult cercopithecoids (Table 4) to ascertain whether the difference between the original and retrodeformed specimens was within the range of variation that would be expected for a single taxon.

**Table 3:** Number of trials per user used to generate the range of variability around intraobserver error.

User	Trials	*D*
AR	3	0.0183
BW	10	0.0163
CS	10	0.0184
MM	10	0.0142
MS	10	0.0133
PW	13	0.0174
TP	5	0.0081
TT	6	0.0166
SF	8	0.0112

*d* indicates the average Procrustes distance between replicates for each user.

**Table 4:** Comparative sample of papionins used in these analyses.

Genus	Females	Males	N
*Cercocebus*	23	31	54
* C. galeritus agilis*	9	9	18
* C. torquatus atys*	2	3	5
* C. t. lunulatus*	1		1
* C. t. torquatus*	11	19	30
*Lophocebus*	22	32	54
* L. aterrimus aterrimus*	4	2	6
* L. albigena johnstoni*	16	28	44
* L. albigena albigena*	2	2	4
*Macaca*	96	117	213
* M. arctoides*	1	3	4
* M. assamensis*	2		2
* M. brunnescens*	3	1	4
* M. cyclopis*		2	2
* M. fascicularis*	22	31	53
* M. fuscata*	3	10	13
* M. hecki*	11	12	23
* M. maura*	1	4	5
* M. mulatta*	15	13	28
* M. nigra*	6	3	9
* M. nemestrina*	7	9	16
* M. radiata*		1	1
* M. silenus*	1		1
* M. sylvanus*	13	15	28
* M. thibetana*	1	2	3
* M. tonkeana*	10	11	21
*Mandrillus*	29	49	78
* M. leucophaeus*	15	26	41
* M. sphinx*	14	23	37
*Papio hamadryas*	176	314	490
* P. h. anubis*	59	125	184
* P. h. hamadryas*	4	30	34
* P. h. kindae*	22	19	41
* P. h. cynocephalus*	8	23	31
* P. h. ursinus*	83	117	200
*Theropithecus gelada*	13	27	40
*Parapapio*	4	3	8 (1 sex unknown)
* P. broomi*	4		4
* P. jonesi*		1	1
* P. whitei*		2	2
* P. sp.*			1 (sex unknown)
*Procercocebus antiquus*		1	1
*Paradolichopithecus arvernensis*	1	3	4

Procrustes distances between the reflected & averaged models and the original cranium were also calculated using both landmark configurations. If the retrodeformed specimens using algorithmic symmetrization had a smaller Procrustes distance to the original specimen than the reflected & averaged models, we can conclude that our method of retrodeformation performed better than simple reflection & averaging in that case.

### Principal components analysis

In order to better visualize how the deformed and retrodeformed crania differ in shape as compared to the original cranium, two principal component analyses (PCAs) including the original cranium, the deformed crania, the algorithmically symmetrized models and the reflected & averaged models were performed. The first PCA uses the Procrustes aligned coordinates for both landmarks and curves, and the second only type I-III landmarks. Retrodeformed models that fall near to the original cranium in the PCA graph would be most similar in shape to the original based on the aspects of shape with the greatest variance in the sample.

To evaluate our reconstructions in a different manner, a PCA of type I, II and III landmark coordinates superimposed by generalized Procrustes analysis (GPA) was also performed on a larger sample of adult male and female *Papio* in order to visualize the position of each algorithmically symmetrized cranium relative to the distribution of *Papio h. kindae* in shape space. If the algorithmically symmetrized crania fall within the expected distribution of a single taxon, then even if the retrodeformation doesn’t perfectly replicate the original, it would still represent a reasonable reconstruction of a member of that taxon.

### Real-world Test Case: *Paradolichopithecus*

**Figure 3:**
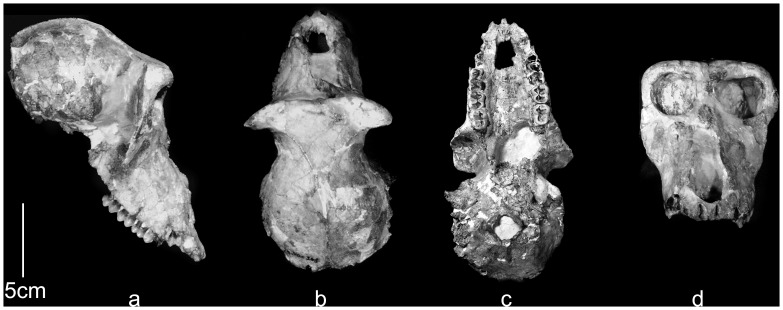
ISER VGr/345 in (a) lateral, (b) superior, (c) basal and (d) anterior views. (a) in approximate Frankfurt horizontal; (b-d) occlusal plane horizontal.

Finally, as a test-case, algorithmic symmetrization was applied to a fossil cranium of a male individual of the Plio-Pleistocene papionin *Paradolichopithecus arvernensis,* from Graunceanu, Romania [30], with landmarks placed on its algorithmically symmetrized surface in Landmark Editor. Special permits were not required to study this specimen. This specimen (ISER [Institute of Speleology Emil Racovitsa, Bucharest, Romania] VGr/345) shows modest but notable asymmetrical deformation (see Figure 3). We chose this *Paradolichopithecus* cranium because it is deformed in a manner typical of many fossil primates and there are several other relatively complete crania (ISER VGr/346, LPB [Laboratory of Paleontology, University of Bucharest, Bucharest, Romania] 300 and FSL [Département des Sciences de la Terre, Université Claude Bernard-Lyon I, Villeurbanne, France; ex. Faculté des Sciences, Lyon] 41333) to which we can compare our exemplar specimen. In all cases the original specimens were landmarked with a Microscribe (by S.R.F.). Only landmarks present in all specimens of our *Paradolichopithecus* sample were utilized in this test case analysis (Fig. 3; Table 1–2). As it is impossible know exactly what ISER VGr/345 looked like prior to diagenesis, we were not able to run the Procrustes distance based assessments, but the retrodeformed fossil was placed in a PCA of all Procrustes aligned coordinates of all extant papionins and fossil *Paradolichopithecus* (Table 4) in order to visually assess the effect of algorithmic symmetrization on its position in shape space relative to the other specimens of *Paradolichopithecus* and other extant and fossil papionins.

## Results

### Tests of artificial deformation: Procrustes Distances

#### CRANIUM 1

**Figure 4:**
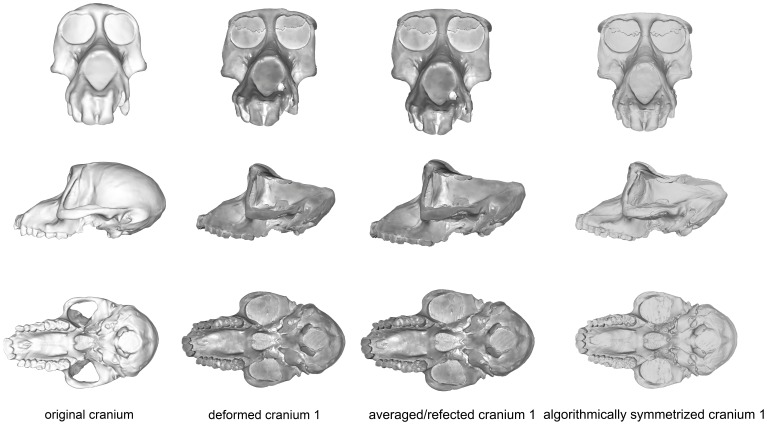
Comparison of the original cranium (left column), deformed cranium 1 (second column), reflected & averaged cranium 1 (third column) and algorithmically symmetrized cranium 1 (right column) in anterior (top), lateral (middle) and basal (bottom) views. Reflected & averaged specimens do not appear perfectly symmetrical as only bilateral landmark points were used in this computation, rather than semilandmark curves or patches.

**Figure 5:**
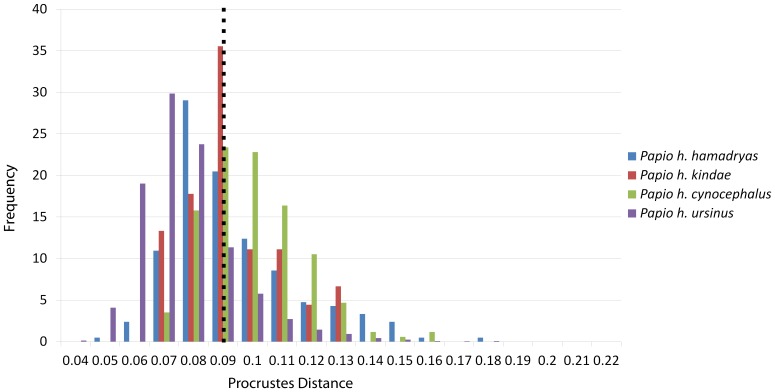
Histogram illustrating the distribution of pairwise Procrustes distances within each group of *Papio*. The dashed line represents the mean within-group pairwise distance for all groups.

**Figure 6:**
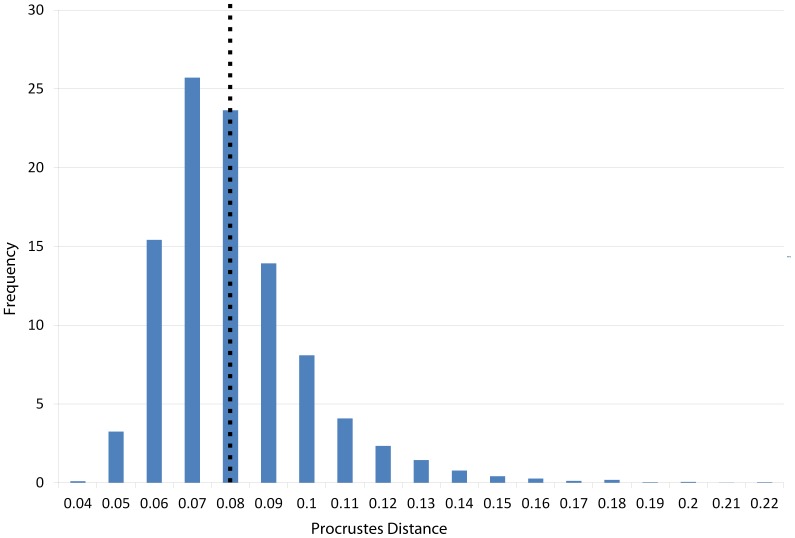
Histogram illustrating the distribution of all pairwise Procrustes distances within each cercopithecid group. Dashed line represents the mean intraspecific pairwise Procrustes distance for all cercopithecids.

**Figure 7:**
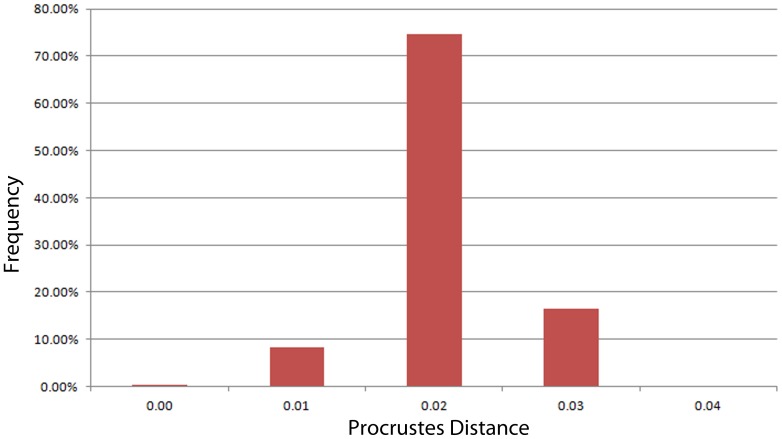
Histogram illustrating the distribution of pairwise Procrustes distances in a study of intraobserver error.

Cranium 1 was the least deformed of the five test crania, as measured by Procrustes distance (Fig. 4; Table 5), with the degree of deformation well within the magnitude of variation of *Papio* (Fig. 5) and all cercopithecoids (Fig. 6). Moderate shear was applied to the entire cranium near the sagittal plane. Algorithmic symmetrization mitigated that shear, and subsequent reflection replaced teeth that are missing on the right side of the original cranium. The Procrustes distance between the algorithmically symmetrized cranium and the original cranium does not improve upon the original pairwise distance (Table 5), although visual assessment shows an improvement in the facial symmetry in areas not covered by semilandmarks. The Procrustes distance between the algorithmically symmetrized cranium 1 and the original cranium is outside the range of intraobserver error (Fig. 7). Reflection & averaging appears to perform equally well in this case (Table 4), and the Procrustes distance between the reflected & averaged model and the original specimen is equal to that of the algorithmically symmetrized model and original – if curves are included – or slightly better, if only type 1, 2 and 3 landmarks are included (Table 5).

**Table 5:** Procrustes distances between the original undeformed cranium and each of the five manual deformations (original to deformed column), as well as their modifications that were reflected & averaged bilaterally (original to reflected & averaged column), and algorithmically symmetrized model (original-retrodeformed).

	Trial	Original todeformed	Original to reflected& averaged	% improvement	Original to algorithmicsymmetrization	% improvement
Including curves	Cranium 1	0.07	0.07	0.0%	0.08	−14.2%
	Cranium 2	0.14	0.12	14.3%	0.10	28.6%
	Cranium 3	0.21	0.08	61.9%	0.08	61.9%
	Cranium 4	0.21	0.16	23.8%	0.19	9.5%
	Cranium 5	0.30	0.19	36.7%	0.11	63.3%
Excluding curves	Cranium 1	0.09	0.08	11.1%	0.08	11.1%
	Cranium 2	0.11	0.06	45.5%	0.10	9.09%
	Cranium 3	0.14	0.11	21.4%	0.09	35.7%
	Cranium 4	0.25	0.12	44.0%	0.15	40.0%
	Cranium 5	0.28	0.20	28.6%	0.15	46.4%

% improvement indicates the percent closer in shape the reflected & averaged model and algorithmically symmetrized model are to the original. The first values are for the landmark configuration including semilandmark curves (landmarked by A.M.). The second set of values are for the landmark configuration without curves (landmarked by S.R.F.).

#### CRANIUM 2

**Figure 8:**
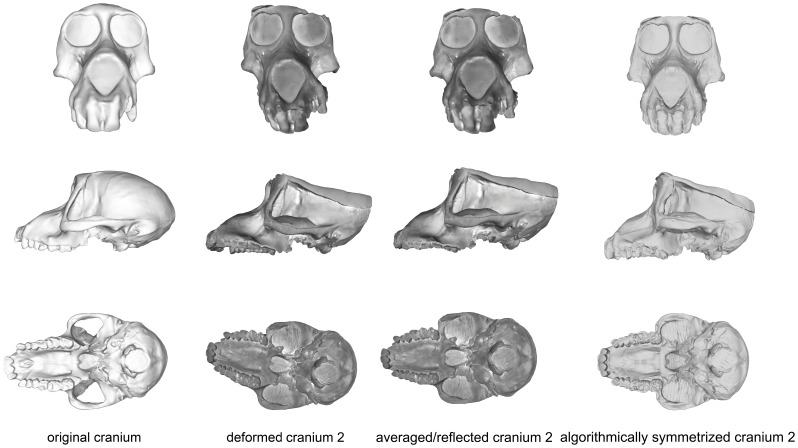
Comparison of the original cranium (left column), deformed cranium 2 (second column), reflected & averaged cranium 2 (third column) and algorithmically symmetrized cranium 2 (right column) in anterior (top), lateral (middle) and basal (bottom) views. Reflected & averaged specimens do not appear perfectly symmetrical as only bilateral landmark points were used in this computation, rather than semilandmark curves or patches.

The maxillary region of cranium 2 was twisted to the left during the deformation process, as seen in the frontal and basicranial views in Figure 8. This twisting also resulted in an anteroposterior shortening of the palate and snout. Algorithmic symmetrization restored symmetry to the face and realigned the face with the neurocranium, while reflection of the retrodeformed cranium replaced the teeth missing in the original cast. However, as the anteroposterior shortening of the palate was a symmetrical deformation, the resulting retrodeformed cranium retains the shortened palate. Reflection & averaging was also able to realign the axis of symmetry and mostly corrected the torsion of the maxillary region. The resultant version is slightly more distant from the original specimen than the algorithmically symmetrized version if curves are included, but more similar to the original if curves are excluded (Table 5). The Procrustes distances from both of the retrodeformed crania, as well as the deformed cranium, to the original specimen were within the magnitude of shape variation expected for *Papio* and all cercopithecoids (Figs. 5–6), but outside the range of intraobserver error (Fig. 7).

#### CRANIUM 3

**Figure 9:**
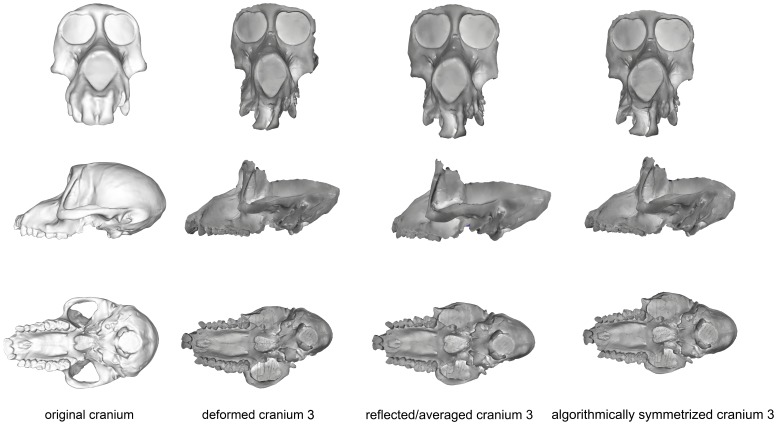
Comparison of the original cranium (left column), deformed cranium 3 (second column), reflected & averaged cranium 3 (third column) and algorithmically symmetrized cranium 3 (right column) in anterior (top), lateral (middle) and basal (bottom) views. Reflected & averaged specimens do not appear perfectly symmetrical as only bilateral landmark points were used in this computation, rather than semilandmark curves or patches.

Deformation was applied asymmetrically to the occipital region of this specimen by depressing only the right side, and the neurocranium was bent slightly relative to the face. The palate was also bent away from the axis of symmetry (Fig. 9). After algorithmic symmetrization, the Procrustes distance to the original cranium was within the range of pairwise Procrustes distances in both *Papio* and all cercopithecoids (Figs. 5–6; Table 5), but outside the range of intraobserver error (Fig. 7). This retrodeformation technique adequately fixed the orientation of the face with respect to the neurocranium, straightened the palate and partially unbent the occipital deformation (Fig. 9). However, the reorientation of the maxilla resulted in a slightly more distorted nasal aperture shape. Reflection & averaging was unable to fully realign the axis of symmetry, and, in addition, the occipital region is narrower than that of the retrodeformed version. If curves are included, reflecting and averaging performs as well as algorithmic symmetrization; if curves are excluded, algorithmic symmetrization performs better (Table 5).

#### CRANIUM 4

**Figure 10:**
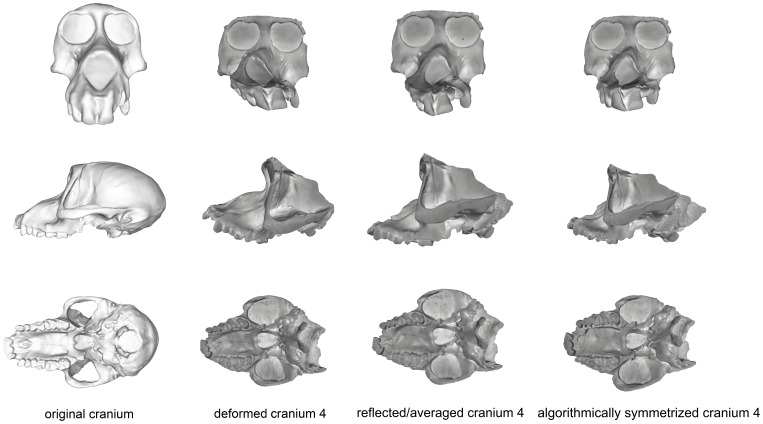
Comparison of the original cranium (left column), deformed cranium 4 (second column), reflected & averaged cranium 3 (third column) and algorithmically symmetrized cranium 4 (right column) in anterior (top), lateral (middle) and basal (bottom) views. Reflected & averaged specimens do not appear perfectly symmetrical as only bilateral landmark points were used in this computation, rather than semilandmark curves or patches.

Cranium 4 was deformed by flattening the maxillary region while pulling it superiorly and pushing anteriorly in the occipital region of the neurocranium (Fig. 10). As most of this deformation was symmetrical, algorithmic symmetrization was not successful in restoring the specimen’s original shape and the Procrustes distance between the original and algorithmically symmetrized crania are larger than the variability contained within an extant species (Figs. 5–6) and far exceeds the maximum intraobserver error (Fig. 7). While symmetry was restored to the palate and maxilla, it could not be restored to its original supero-inferior height, which remained shallower than the original, as that deformation was symmetrical. Similarly, while the occipital region was symmetrized, it could not be re-inflated to match the original. Reflection & averaging also performed poorly for this cranium, with the resultant model retaining the same errors as the algorithmically symmetrized model but with less symmetry. In comparison with the algorithmically symmetrized version, the reflected & averaged version has a less upturned maxillary region, and the palate is longer. For both landmark configurations, reflection & averaging improved upon the resulting shape more than algorithmic symmetrization (Table 5).

#### CRANIUM 5

**Figure 11:**
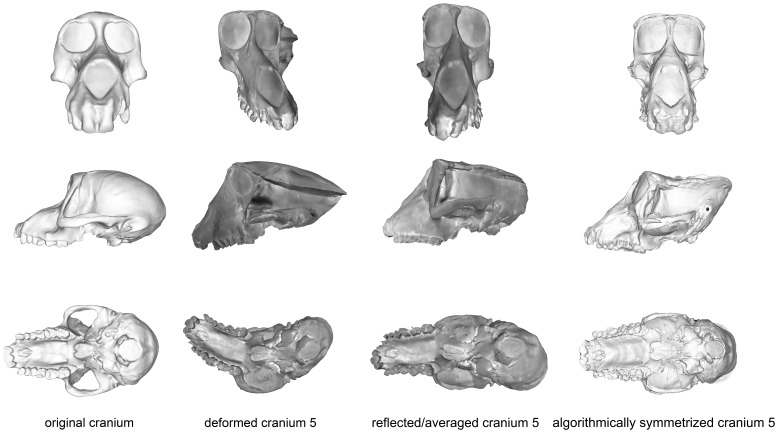
Comparison of the original cranium (left column), deformed cranium 5 (second column), reflected & averaged cranium 5 (third column) and algorithmically symmetrized cranium 5 (right column) in anterior (top), lateral (middle) and basal (bottom) views. Reflected & averaged specimens do not appear perfectly symmetrical as only bilateral landmark points were used in this computation, rather than semilandmark curves or patches.

This specimen represents the most extreme deformation from the original cranium as measured by Procrustes distance from the original (Fig. 11; Table 5). In this test, the entire cranium was both mediolaterally squeezed and anteroposteriorly bent to the left. The algorithmic symmerization improved upon the Procrustes distance to the original by over 40% (Table 5). In particular, the algorithmically symmetrized cranium was properly realigned in an anteroposterior direction. However, as the mediolateral pinching was more symmetrical, the algorithmically symmetrized cranium is narrower than the original. Additionally, the occipital region is more steeply angled than in the original cranium due to the more symmetric squeezing in the deformation. Despite the deformation in cranium 5 being greater than that of cranium 4, the symmetrization algorithm was able to make greater improvement on this cranium (Table 5), as more of the deformation was asymmetric, and the Procrustes distance between the resulting model and original cranium is within the intraspecific variability in *Papio* (Fig. 5) and all cercopithecoids (Fig. 6), although outside the range of intraobserver error (Fig. 7). Reflection & averaging performed poorly in comparison. This technique was unable to completely unbend the face or restore the zygomatic arch on the right side to its original form. The occipital bone in this model is rounded more appropriately, but the foramen magnum appears oval rather than round. Regardless of the landmark configuration, the Procrustes distance between this model and the original specimen is greater than the algorithmically symmetrized version (Table 5).

### Tests of artificial deformation: PCAs

**Figure 12:**
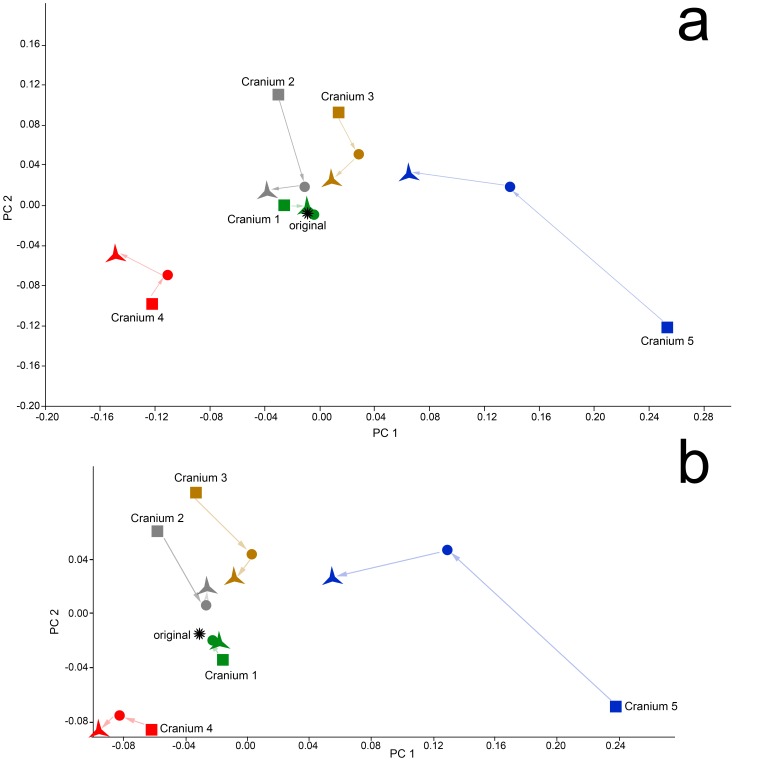
PCA of the Procrustes aligned coordinates for the original (star), deformed (squares), reflected & averaged (circles) and algorithmically symmetrized (triangles) crania. Arrows connect the deformed to the reflected & averaged model, and the reflected & averaged model to the algorithmically symmetrized cranium. These arrows are for aid in visualization and do not represent real data. (a) PCA including both semilandmark curves and type I, II and III landmarks. PC1 accounts for 49% and PC 2 18% of the variance within this sample. (b) PCA of the Procrustes aligned coordinates including only types I-III landmarks. PC 1 accounts for 52% and PC 2 20% of the variance within this sample.

PCAs of the deformed, retrodeformed and original crania are presented in Figure 12 and illustrate the results of Table 5. For the landmark set including semilandmarks (Fig. 12a), the algorithmically symmetrized versions of crania 3 and 5 are clearly closest to the original specimen in the combined shape space of principal components (PC) 1 and 2. The mirrored and averaged cranium 2 and algorithmically symmetrized cranium 2 are both close to the original specimen, but occupy slightly different places in shape space. Both the algorithmically symmetrized cranium 1 and mirrored and averaged cranium 1 are virtually identical to the original specimen. Only in the case of cranium 4 is the algorithmically symmetrized version farther away from the original than the reflected & averaged version. The results of this analysis utilizing only type I-III landmarks are similar for all crania except cranium 2; when semilandmarks are removed from the analysis, the reflected & averaged cranium 2 is closer to the original than the algorithmically symmetrized version, indicating that the Type 1–III landmarks alone do not capture as much anatomical detail. All of these results echoed the results of the tests using Procrustes distances (Table 5).

**Figure 13:**
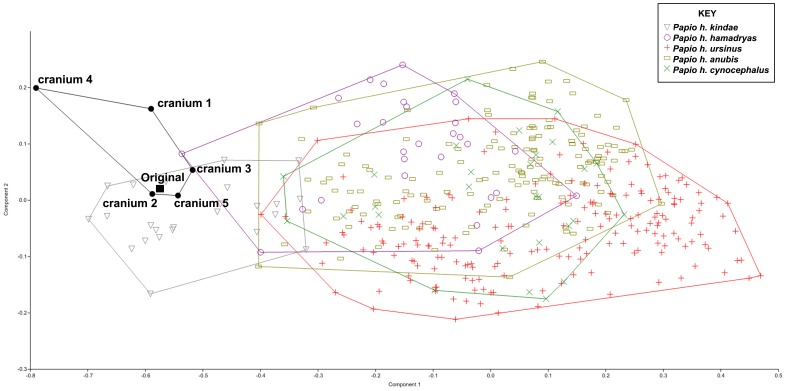
PCA of the algorithmically symmetrized specimens with the full sample of *Papio*. Landmarks 1–3, 13–15, and 24–27 were eliminated from the original dataset to accommodate the retrodeformed specimens. Specimens are labeled in the graph as per the key. Lines represent convex hulls surrounding each genus.

The result of a PCA of the deformed and algorithmically symmetrized crania with a large sample of *Papio* crania is presented in Figure 13. While none of the algorithmically symmetrized crania were exactly the same as the original cranium, three out of five crania fell within the convex hull for *Papio h. kindae,* and of the two that fell outside that convex hull, cranium 3 was inside the *P.h. kindae* distribution on PC 1. Cranium 4 was farthest away from the cluster, falling outside the distribution of *P.h. kindae* on PC 1 and 2, which was expected as it has the greatest degree of uncorrected symmetrical deformation.

### *Paradolichopithecus arvernensis*, as a test case

**Figure 14:**
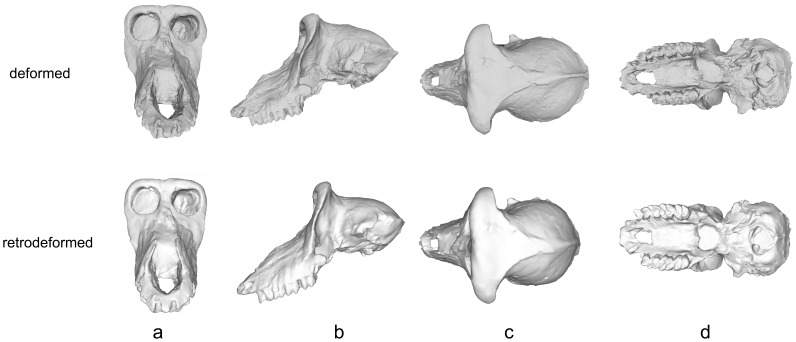
Deformed and algorithmically symmetrized scans of ISER VGr/345 (*Paradolichopithecus arvernensis)* in (a) anterior, (b) lateral, (c) superior and (d) basal views.

**Figure 15:**
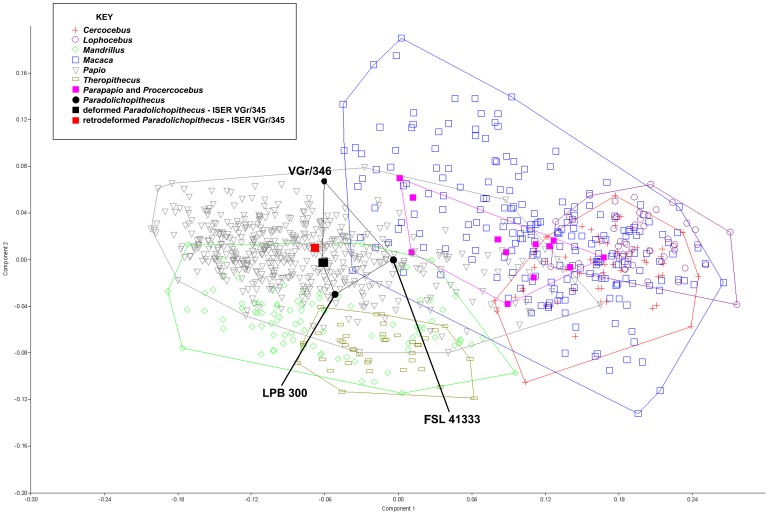
PCA of all extant cercopithecid taxa, *Paradolichopithecus,* and algorithmically symmetrized *Paradolichopithecus*. Specimens are labeled in the graph as per the key. Lines represent convex hulls surrounding each genus.

The cranium of ISER VGr/345 was subjected to diagenetic change during the process of fossilization. Manual preparation of the specimen under the direction of E.D. was partly able to correct more extensive deformation, but the “offset” between the face and the palate could not be repaired. In addition, the left side of the cranium remains sheared inferiorly, and there is a distinct bend between the face and the neurocranium, especially in inferior view (Fig. 3, 14). These types of real deformations are similar to the manufactured deformations in crania 2 and 3 (Figs. 8–9). Algorithmic symmetrization of VGr/345 restores symmetry to the face and realigns the face with the neurocranium (Fig. 14). It can be compared to three other specimens of the same species: FSL 41333, the holotype, is a female cranium (from the slightly younger locality of Seneze, France) manually reconstructed from numerous undeformed fragments; VGr/346 is a large minimally deformed male face lacking the entire neurocranium; LPB 300 is a male in which the face was mostly reconstructed manually and the neurocranium restored in plaster on the basis of VGr/345 (the latter two are from Graunceanu, Romania, the same locality as VGr/345). A PCA of all papionins, including those three specimens of *Paradolichopithecus* and both the original and algorithmically symmetrized versions of VGr/345, is presented in Fig. 15. The algorithmically symmetrized VGr/345 falls close to the deformed original on PC 1 and slightly closer to VGr/346 on PC 2. The algorithmically symmetrized specimen is also most similar in shape to other *Paradolichopithecus* specimens as measured by Procrustes distance (Table 6), although more dissimilar in shape to all of the papionin taxon means and other *Paradolichopithecus* specimens.

**Table 6:** Procrustes distances between the retrodeformed *Paradolichopithecus* specimens, the other *Paradolichopithecus* individuals and the extant taxon means; details about extant species in Table 4.

	ISER VGr/345	FSL 41333	LPB 300	VGr/346	*Cerc.*	*Mac.*	*Loph.*	*Man.*	*Thero.*	*Para.*	*Pap.*
FSL 41333	0.11										
LPB 300	0.08	0.13									
VGr/346	0.09	0.12	0.11								
*Cercocebus*	0.18	0.17	0.16	0.21							
*Macaca*	0.19	0.15	0.19	0.19	0.16						
*Lophocebus*	0.28	0.24	0.28	0.29	0.18	0.21					
*Mandrillus*	0.12	0.15	0.13	0.17	0.18	0.24	0.28				
*Theropithecus*	0.15	0.17	0.14	0.18	0.21	0.24	0.30	0.16			
*Parapapio/Procercocebus*	0.25	0.22	0.24	0.26	0.17	0.20	0.14	0.26	0.26		
*Papio*	0.09	0.13	0.11	0.11	0.21	0.23	0.31	0.11	0.16	0.28	
**Algorithmically symmetrized Paradolichopithecus**	**0.09**	**0.13**	**0.11**	**0.12**	**0.20**	**0.19**	**0.30**	**0.16**	**0.19**	**0.27**	**0.12**

## Discussion

### Tests of retrodeformation

Diagenetic change during the process of fossilization can result in a nearly infinite number of distortions, of which symmetrical deformation is the most challenging to correct [5]. This is true not only for the algorithmic symmetrization technique presented here, but for all currently employed symmetrization approaches [5],[8],[9]. If the original deformation is symmetrical, then that deformation will still be present to some degree in the retrodeformed result. Of the five mechanically distorted crania, our algorithmic symmetrization technique removed the smallest amount of deformation, as measured by our Procrustes distance test, when applied to cranium 4 (Fig. 10). This is because of the symmetrical nature of the distortion of that specimen: both the supero-inferior compression of the most anterior aspect of the maxillary region and the antero-posterior compression of the most posterior portion of the occipital region. In order to fully retrodeform a specimen that has been subjected to symmetrical deformation, extra steps would need to be performed, such as comparing the distorted specimens to appropriate extant or less distorted fossil individuals to estimate the degree of affine stretch to apply.

Asymmetrical deformation is better handled by all symmetrization-based techniques [4], [8], [9]. The analyses presented here illustrate that the algorithmic symmetrization technique of Ghosh et al. [9] handles asymmetric deformation and performs particularly well when the original deformation involves shearing and bending. In three of the test crania, the algorithmically symmetrized versions were within the expected distribution of the species on which they were based, lending support to the idea that while algorithmically symmetrized specimens may not be perfect replicas of the original, they are a reasonable representation of a member of their taxon.

These analyses also demonstrate that the algorithmic symmetrization technique represents an improvement on what is possible with simple reflection & averaging when the original deformation is great. In the most deformed cranium (5), algorithmic symmetrization far outperformed reflection & averaging for restoring the specimen to its original shape. At smaller levels of deformation, reflection & averaging and algorithmic symmetrization performed equally well. Reflection & averaging only performed substantially better in the retrodeformation of Cranium 4. The greatest difference between the two results is in the shape of the maxilla: with reflection & averaging, it was possible to angle the maxilla to a position that more closely matched that of the original cranium; however, perhaps with a different selection of landmarks the method of algorithmic symmetrization could perform equally well. For Cranium 2, reflection & averaging performed better than algorithmic symmetrization when curves were removed from the analysis. This is likely because the type I, II, and III landmarks alone do not capture the geometry of the maxilla as well as do curves.

Given that the goal of this paper was to objectively evaluate the performance of different ways of restoring symmetry, the results presented here were not based on complete retrodeformations, but only implemented the symmetrization component. In order to fully restore these specimens, missing parts would need to be replaced or imputed; other processes, such as refitting displaced but otherwise intact components, would improve these results further. Having demonstrated the efficacy of the method, especially with extremely distorted specimens, we aim to use it in conjunction with these additional steps to restore additional fossils. We will also provide scans of the original and deformed specimens to interested colleagues so that our several methods of retrodeformation can be compared objectively; such collaboration may lead to improved methods combining different approaches. In addition, the plugin for Landmark Editor for retrodeformation by algorithmic symmetrization is freely available at http://www.cs.ucdavis.edu/~amenta/retrodef.html.

### Algorithmic symmetrization of *Paradolichopithecus*

ISER V/Gr 345 is a lightly deformed *Paradolichopithecus* specimen. Considering this, it is perhaps not surprising that there was little difference in its placement with respect to the other specimens of *Paradolichopithecus* in a PCA (Fig. 15). However, in order to rigorously test our methodology on a fossil individual, it was essential to choose a species that is reasonably well-represented in the fossil record with multiple securely identified crania. Algorithmic symmetrization had the effect of moving ISER VGr/345 away from LBP 300 and closer to VGr/346. LBP 300 has been largely reconstructed by hand using plaster whereas VGr/346 is mostly intact. Algorithmic symmetrization also had the effect of making the retrodeformed version of ISER VGr/345 less like all of the papionin taxon means and other *Paradolichopithecus* specimens. This is likely because the retrodeformed *Paradolichopithecus* specimen is perfectly symmetrical whereas the other *Paradolichopithecus* specimens and the papionin taxon means are not.

## Summary and Conclusions

We mechanically deformed five casts of a cranium of *Papio hamadryas kindae* of known shape and retrodeformed them using both reflection & averaging and algorithmic symmetrization. Our results indicate that algorithmic symmetrization represents a significant improvement over reflection & averaging when distortion is relatively large and asymmetrical. Here we do not present completed retrodeformations, but rather evaluate the symmetrization component of the larger retrodeformation process; this suggests that algorithmic symmetrization should be implemented, along with the other known tools of retrodeformation, to yield improved reconstructions of fossil specimens [4]. The use of manually deformed versions of known-morphology specimens provides a means of testing the quality of the result. The application of our algorithmic symmetrization approach to a real fossil of *Paradolichopithecus arvernensis* resulted in a small but significant improvement to the symmetry of a manually-reconstructed specimen.
